# Single-center experience description of surgical management of diffuse congenital hyperinsulinism in a pediatric non-current cohort

**DOI:** 10.3389/fendo.2026.1760749

**Published:** 2026-03-23

**Authors:** Xiaoxiang Li, Congli Chen, Yangmingyue Ji, Yuxi Wang, Yanmei Sang

**Affiliations:** 1 Department of Endocrinology, Genetics and Metabolism, Beijing Children’s Hospital, Capital Medical University, National Center for Children’s Health, Beijing, China; 2 Department of Pediatrics, Beijing Luhe Hospital, Capital Medical University, Beijing, China; 3 Department of Pediatrics, West China Second University Hospital, Sichuan University, Chengdu, China; 4 Key Laboratory of Birth Defects and Related Diseases of Women and Children, Ministry of Education, West China Second University Hospital, Sichuan University, Chengdu, China

**Keywords:** ABCC8 protein, congenital hyperinsulinism, diffuse, KCNJ11 protein, neurodevelopmental disorders, pancreatectomy, pancreatectomy outcomes, study cohort

## Abstract

**Introduction:**

In this study, we describe surgical indications, procedure selection, and postoperative outcomes in Chinese children with diffuse congenital hyperinsulinism (DCHI). Pancreatic involvement was assessed using ^18^F-L-Fluoro-3,4-dihydroxyphenylalanine positron emission tomography/computed tomography (^18^F–L–DOPA PET/CT) to support clinical decision-making.

**Methods:**

Clinical and genetic characteristics were summarized for 27 children with DCHI, stratified into a surgical group (*n* = 12) and a non-surgical group (*n* = 15). Clinical characteristics were summarized descriptively. An exploratory Firth penalized logistic regression was applied to illustrate descriptive patterns in clinical features considered during surgical intervention selection at this center, without implying causal relationships or predictive effects. Metabolic outcomes after subtotal pancreatectomy (STP) and near-total pancreatectomy (NTP) were compared descriptively. Median postoperative follow-up was 2.9 years (range 2.0–8.8 years). Neurodevelopmental outcomes were assessed using standardized tools at variable ages. Kaplan–Meier analysis was applied descriptively to illustrate long-term neurological trends.

**Results:**

Of the 27 patients with medically refractory diffuse congenital hyperinsulinism, 12 (44.4%) required surgical intervention, including subtotal pancreatectomy (STP, n = 6) and near-total pancreatectomy (NTP, *n* = 6). The median age at diagnosis was 3 days (IQR 2–7), with a male-to-female ratio of 1.25:1. Among the 12 surgical patients, 8 underwent genetic testing, and all (8/8, 100%) were found to harbor ABCC8/KCNJ11 mutations. Overall hypoglycemia control patterns were comparable between the NTP and STP groups, although distinct long-term metabolic trade-offs were observed. At the final follow-up, postoperative diabetes (PD) was observed in 16.7% of NTP patients, while long-term neurodevelopmental outcomes were comparable between the surgical and non-surgical groups.

**Conclusions:**

Subtotal and near-total pancreatectomy were not observed to result in major perioperative complications in this cohort of Chinese pediatric patients with medically refractory DCHI. Clinicians may need to carefully balance hypoglycemia control against the frequency of postoperative diabetes when selecting the extent of resection. Early diagnosis, timely hypoglycemia management, and structured long-term follow-up are essential to optimize long-term outcomes.

## 1 Introduction

Congenital hyperinsulinism (CHI) is one of the most common causes of persistent hypoglycemia in infants and children (1). This genetically heterogeneous disorder exhibits an incidence of approximately 1 in 25,000–50,000 live births (2), rising to around 1 in 2,500 in populations with a high rate of consanguinity (3). The principal risk associated with CHI results from inappropriate and excessive insulin secretion, leading to recurrent and prolonged hypoglycemia that may contribute to irreversible brain injury during the neonatal period. Neurological complications may include seizures, developmental delay, intellectual disability, and cerebral palsy (4, 5). Thus, timely diagnosis and intervention are important to mitigate long-term neurodevelopmental sequelae.

Given the rarity and clinical heterogeneity of CHI, evidence is largely derived from small case series and single-center experiences. Accordingly, this study was designed as a descriptive, exploratory analysis to provide preliminary insights into real-world surgical decision-making, procedural selection, and long-term outcomes in children with medically refractory diffuse CHI.

Clinical management is primarily pharmacological, with diazoxide as the first-line treatment, and octreotide and lanreotide as second-line options (6–8). Sirolimus has been considered for selected refractory cases (9), but its use remains controversial and is not universally recommended. Previous studies have reported considerable variability in diazoxide responsiveness, with approximately 25%–30% of patients showing no response (10). Among these medically refractory cases, the proportion of diffuse CHI (DCHI) is thought to be relatively high (30%–50%), particularly in patients harboring mutations in the ATP-binding cassette subfamily C member 8 (*ABCC8*) and Potassium inwardly rectifying channel subfamily J member 11 (*KCNJ11*) genes, who often require surgical intervention according to several clinical cohorts (11).

Based on histopathology, CHI is classified into focal, diffuse, and atypical forms, reflecting substantial clinical and histological heterogeneity (12–14). Among these, CHI resulting from mutations in genes encoding the ATP-sensitive potassium (K_ATP_) channel is considered the most common and one of the most severe subtypes, with the diffuse form representing a substantial proportion of K_ATP_ mutation–associated CHI cases (15). Unlike the focal form, which can be cured by precise surgical resection, DCHI involves the entire pancreas, posing considerable clinical challenges. Identifying the optimal surgical approach and predicting long-term prognosis remains complex (6).

Surgical management of DCHI typically involves subtotal pancreatectomy (approximately 80–94% of the gland) or near-total pancreatectomy (95–98%) (6). Near-total resection has been reported in some studies to be associated with improved hypoglycemia control, although postoperative diabetes (PD) is frequently observed, with some series reporting rates approaching 100% (16). Conversely, recurrent hypoglycemia appears to be more common following subtotal pancreatectomy, with relapse rates reported as high as 50% (17), and some patients subsequently may require reoperation. Balancing postoperative glycemic control with the risk of PD, defining surgical indications, and selecting the extent of resection remain critical considerations. Furthermore, long-term neurological outcomes are largely described descriptively rather than inferentially.

Although international guidelines provide important references for CHI management, data on surgical outcomes in Chinese children with diffuse congenital hyperinsulinism remain limited.

This single-center, retrospective cohort study aims to provide descriptive insights into real-world institutional practice patterns in surgical decision-making, procedural selection, and long-term outcomes in pediatric patients with medically refractory diffuse congenital hyperinsulinism. All analyses were descriptive and exploratory and were not intended to imply treatment efficacy, establish causality, or support predictive inference. Specifically, Kaplan–Meier curves were applied to describe overall patterns of long-term outcomes, rather than to draw inferential conclusions.

## 2 Materials and methods

### 2.1 Study design and patient enrollment

This single-center, retrospective cohort study describes a series of children with diffuse congenital hyperinsulinism (DCHI) treated at Beijing Children’s Hospital between 2002 and 2023. A total of 27 pediatric patients with medically refractory DCHI were included in this retrospective cohort. Patients were classified according to treatment exposure (surgical or non-surgical), and clinical outcomes were assessed during follow-up. To ensure accurate histopathological classification and to exclude focal lesions, all diffuse cases were confirmed using ^18^F**–**L**–**DOPA PET/CT scanning (18).

This study was designed as a descriptive, retrospective cohort analysis. Accordingly, all comparisons and statistical models were applied within an exploratory, hypothesis-generating framework; they were not intended to evaluate treatment efficacy or establish causality.

### 2.2 Cohort stratification

Patients were stratified according to treatment strategy: the surgical group (*n* = 12), defined as those with medically refractory DCHI (see Section 2.3.2 for criteria), all of whom underwent pancreatic resection, and the non-surgical group (*n* = 15), consisting of patients who achieved stable glycemic control with medical therapy and did not meet the criteria for surgical intervention. The surgical group was further subdivided by extent of resection into the near-total pancreatectomy (NTP, 95%–98%, *n* = 6) and subtotal pancreatectomy (STP, 80%–94%, *n* = 6).

No patient in the non-surgical group underwent surgery during the study period, as they did not meet the predefined criteria. Surgical management was applied only in patients with persistent hypoglycemia despite maximal medical therapy.

### 2.3 Diagnostic and treatment criteria

#### 2.3.1 Diagnostic criteria

CHI was diagnosed according to the international consensus criteria proposed by De Leon et al. (6). Diagnostic criteria included both biochemical and imaging components.

##### 2.3.1.1 Biochemical criteria

Abnormal insulin secretion during hypoglycemia, including plasma insulin > 1.25 μU/mL and C-peptide > 0.5 ng/mL, with concomitant plasma free fatty acids < 1.7 mmol/L and β-hydroxybutyrate < 1.8 mmol/L. Glucose infusion rate (GIR) required to maintain euglycemia was > 8 mg/kg/min in neonates and > 3 mg/kg/min in older children.

Imaging criteria: ^18^F–L–DOPA PET/CT evaluation. DCHI was defined as homogeneous increased uptake throughout the pancreas without focal hotspots (19, 20).

#### 2.3.2 Medical therapy

##### 2.3.2.1 Diazoxide

Initial dose was 5 mg/kg/day, titrated to a maximum of 15 mg/kg/day, combined with hydrochlorothiazide to prevent fluid/sodium retention.

##### 2.3.2.2 Octreotide

Initial dose of 5 μg/kg/day, titrated up to a maximum of 20 μg/kg/day.

Medical refractoriness was defined as failure to maintain fasting blood glucose ≥ 3.0 mmol/L after ≥ 5 days of maximal diazoxide therapy (15 mg/kg/day) and insufficient response to octreotide, or continued need for IV glucose/GIR above age-specific thresholds (21, 22).

Stable glycemic control was defined as fasting blood glucose ≥3.0 mmol/L without intravenous glucose support and without recurrent symptomatic hypoglycemia during routine daily life, consistent with accepted neonatal hypoglycemia thresholds (21, 22).

#### 2.3.3 Surgical procedures

The choice between STP and NTP was not randomized and was not dictated by a predefined temporal change in institutional guidelines. Instead, the surgical strategy was individualized intraoperatively. Decisions were based on frozen section pathology confirming diffuse disease, intraoperative assessment of pancreatic tissue appearance, and the patient’s preoperative refractoriness to medical therapy.

Surgical decision-making involved balancing adequate glycemic control with preservation of pancreatic tissue. The goal was to maximize postoperative euglycemia while minimizing long-term insulin-dependent PD risk. These considerations, together with overall disease severity and intraoperative findings, determined the final extent of resection.

The predefined resection categories (subtotal pancreatectomy, ~80–94%; near-total pancreatectomy, ~95–98%) were based on commonly used definitions in the CHI surgical literature. Near-total resection refers to removal of nearly all pancreatic tissue, leaving a small rim around the duodenum/ampulla, whereas subtotal resection preserves relatively more tissue. In this study, resection percentages were derived from intraoperative estimates recorded in operative reports rather than pathological volumetric quantification.

Histopathological examination confirmed DCHI in all surgical cases, including intraoperative frozen section analysis to guide resection extent. This approach is consistent with definitions used in prior CHI surgical studies and observational reports (CHOP, Beltrand, Adzick) (6, 23, 24), providing descriptive context for our cohort.

#### 2.3.4 Treatment timeline variables

Retrospectively extracted variables: (i) Age at hypoglycemia onset: first documented plasma glucose < 2.6 mmol/L requiring intervention. (ii) Duration of preoperative medical therapy: from initiation of maximal medical therapy to surgery or stable glycemic control. (iii) Surgical decision delay: time from meeting surgical criteria to actual surgery (criteria = persistent hypoglycemia despite ≥ 5 days of maximal diazoxide plus second-line agents) (25). (iv) Age at surgical intervention. Exact cumulative duration of hypoglycemia prior to surgery was not consistently documented; surrogate indicators such as age at surgery and duration of preoperative therapy were used (4).

For patients managed non-surgically, duration of medical therapy was defined as the interval from diagnosis to the achievement of stable glycemic control without IV glucose support.

## 3 Outcome measures

Baseline characteristics, including age at onset, sex, birth weight, blood glucose, insulin, and C-peptide levels, were compared between the surgical and non-surgical groups. Long-term follow-up (2–13 years) was conducted for all patients, and among surgical patients. Surgical outcomes were further stratified by extent of pancreatectomy (subtotal vs near-total). Key endpoints included metabolic outcomes (remission, recurrent/persistent hypoglycemia, and postoperative insulin-dependent diabetes) and neurodevelopmental outcomes.

### 3.1 Clinical outcomes

Long-term follow-up was conducted through outpatient visits at our center and structured telephone interviews for patients who continued follow-up at local hospitals. Follow-up assessments were generally performed at 6 to 12 month intervals, depending on clinical needs and data availability. Owing to the retrospective design of the study and variability in follow-up practices across different institutions, systematic metabolic assessments —including HbA1c, oral glucose tolerance tests (OGTT), and C-peptide measurements—were not routinely available. Postoperative follow-up focused on clinically relevant glycemic endpoints, including normalization of blood glucose levels, recurrence of hypoglycemia, and development of PD. PD was defined as either documented requirement for long-term insulin therapy or physician-diagnosed PD during follow-up. Individual patient follow-up ranged from 2 to 13 years, with visits conducted at our center or via structured telephone interviews for local patients.

Clinical outcomes were evaluated across distinct postoperative metabolic domains, including hypoglycemia remission, persistent or recurrent hypoglycemia, and PD. These outcomes were analyzed and reported separately to avoid conflating persistence of hyperinsulinism with iatrogenic insulin deficiency.

### 3.2 Definitions

Postoperative outcomes and surgical procedures were defined in **Supplementary Table 1**: STP, NTP, postoperative remission, persistent hypoglycemia, and PD.

### 3.3 Neurological sequelae (neurodevelopmental disorders)

Neurological sequelae were assessed based on available clinical follow-up data using standardized psychometric tests (Gesell Developmental Schedules or Wechsler Intelligence Scale for Children) when available, structured milestone-based evaluations when age-appropriate, or structured telephone interviews for patients without formal testing. To account for heterogeneity in assessment tools, outcomes were categorized as normal or delayed. Developmental delay was defined as DQ/IQ < 70 for patients with standardized assessments, or as clinically evident delay based on age-appropriate milestones for children < 6 years, consistent with previous studies (26). Missing data were handled using an available-case approach, and the number of patients lost to follow-up is reported in **Table 1**. Kaplan–Meier analysis was used descriptively to illustrate cumulative neurological risk, accounting for variable follow-up durations and censored observations. Neurodevelopmental assessments were not performed blinded to treatment group due to the retrospective design and the availability of treatment information in clinical records; however, standardized assessment tools were used whenever available to minimize assessment bias.

**Table 1:** Exploratory comparison of metabolic and long-term neurological outcomes between surgical and non-surgical groups in patients with DCHI.

Outcome	Surgical group (*n* = 12)	Non-surgical group (*n* = 15)	*P* value
Favorable neurological outcome, *n/N* (%)	8/11 (72.7%)	8/14 (57.1%)	0.678 *
Neurological outcomes (delay/epilepsy), *n/N* (%)	3/11 (27.3%)	6/14 (42.9%)	0.678 *
Lost to follow-up, *n*	1	1	—

Data are presented as *n* (%). Favorable neurological outcome was defined as normal age-appropriate neurodevelopment without psychomotor delay or epilepsy at last follow-up. Neurodevelopmental delay included psychomotor or cognitive impairment and/or epilepsy. *P* values were calculated using Fisher’s exact test based on patients with available follow-up data; patients lost to follow-up were excluded from outcome analyses. Denominators reflect patients with available long-term neurodevelopmental follow-up.

## 4 Statistical analysis

All analyses were prespecified as exploratory and descriptive, reflecting the non-comparative study design, small sample size, and clinician-driven treatment decisions. Continuous variables are summarized as median (IQR) and compared using the Mann–Whitney *U* test. Categorical variables are presented as counts (*n*, %) and were compared using Fisher’s exact test. Exploratory Firth penalized logistic regression was used descriptively to illustrate institutional patterns in how selected clinical characteristics were considered in decisions regarding surgical intervention within this center. Given the reliance on clinician judgment, institutional practice patterns, and the resulting wide confidence intervals associated with the small sample size, these analyses were performed for descriptive and hypothesis-generating purposes only. No causal relationships or predictive inferences were intended.

Kaplan–Meier curves were applied descriptively to illustrate temporal trends in the neurological sequelae, rather than to support formal inferential comparisons. Although the log-rank test was performed, any resulting P values were interpreted with caution and were not intended to imply statistically robust group differences. Individual patient follow-up ranged from 2 to 13 years. Patients lost to follow-up (one per group) were treated as censored, and Kaplan–Meier analysis was used to visualize long-term outcome patterns in this exploratory cohort. Accordingly, survival analyses were intended to provide descriptive insights into long-term trends rather than definitive estimates of risk or effect. P-values < 0.05 were used for descriptive reference only, without implying formal statistical significance. All analyses were performed using IBM SPSS Statistics (version 27).

### 4.1 Missing data

Baseline demographic data were complete for all patients. However, some biochemical and genetic data were incomplete, including postoperative metabolic markers (e.g., HbA1c, oral glucose tolerance test [OGTT], and C-peptide), and not all patients underwent comprehensive genetic testing. The cumulative duration of hypoglycemia prior to surgery could not be precisely quantified due to the retrospective design. Long-term neurodevelopmental follow-up was available for 25 of 27 patients; one patient in each group was lost to follow-up. Analyses were conducted using an available-case approach without imputation. Given the small and balanced loss to follow-up, its impact on exploratory analyses is expected to be minimal.

## 5 Results

### 5.1 Baseline characteristics of 27 children with diffuse CHI

**Figure 1:**
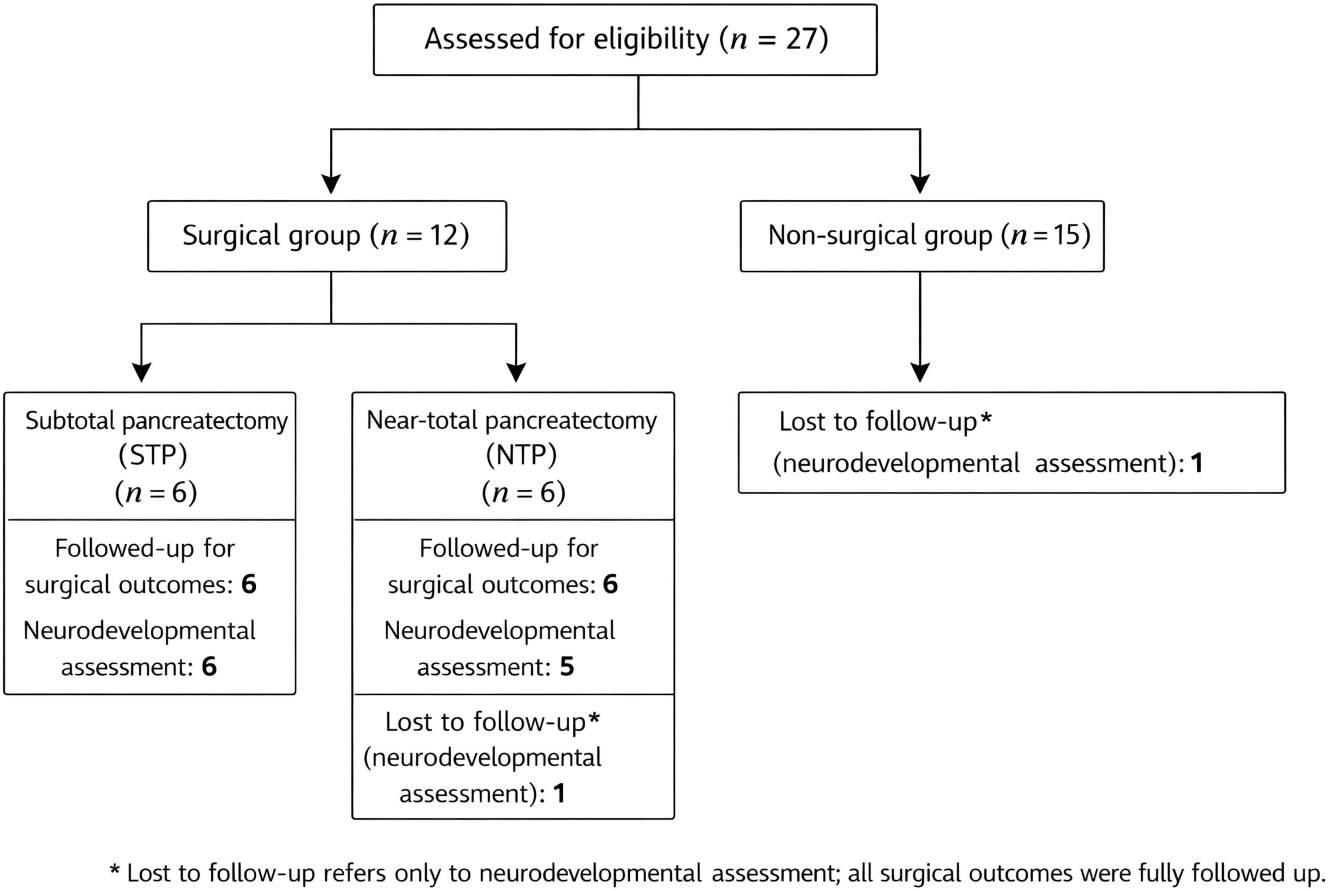
Flow diagram of patient inclusion, treatment allocation, and follow-up. A total of 27 children with diffuse congenital hyperinsulinism (CHI) confirmed by ^18^F–L–DOPA PET/CT were included. Of these, 12 patients underwent surgical treatment (subtotal or near-total pancreatectomy), and 15 were managed without surgery. One patient in each group was lost to neurological follow-up and was treated as censored in the Kaplan–Meier analyses. Surgical outcomes and postoperative complication data were available for all surgically treated patients. * Lost to follow-up refers to neurological outcome assessment only.

A total of 27 children with diffuse CHI confirmed by ^18^F–L–DOPA PET/CT were enrolled. The patient inclusion, treatment allocation, and follow-up processes are summarized in **Figure 1**. Baseline characteristics were generally comparable between the surgical and non-surgical groups (**Table 2**).

**Table 2:** Baseline clinical characteristics of 27 children with diffuse CHI.

Variable	Surgical group (*n* = 12)	Non-surgical group (*n* = 15)	*P* value
Age at onset, days, median (IQR)	0.5 (0–2)	1 (0–150)	0.500
Sex (male/female)	9/3 (75.0%/25.0%)	11/4 (73.3%/26.7%)	0.999
Onset period, *n* (%)	Neonatal: 11 (91.7%) Infant: 0 (0.0%) Childhood: 1 (8.3%)	Neonatal: 9 (60.0%) Infant: 5 (33.3%) Childhood: 1 (6.7%)	0.132
Birth weight < 4 kg, *n* (%) /≥ 4 kg, *n* (%)	3 (25.0%) /9 (75.0%)	8 (53.3%) /7 (46.7%)	0.239
Insulin, mIU/L, median (IQR)	27.10 (14.70–58.28)	16.76 (9.72–41.55)	0.157
C–peptide, ng/mL, median (IQR)	2.94 (1.64–4.64)	2.65 (1.87–6.44)	0.943
Genetic testing status (*n*, tested)			
*ABCC8*/*KCNJ11* mutation	8 (8/8 tested)	7 (2/11 tested)	Not applicable
Other gene mutations	0 (0/8 tested)	2 (2/11 tested)	Not applicable
Mutation-negative (tested)	0 (0/8 tested)	2 (2/11 tested)	Not applicable
Not tested	4 (4/12)	4 (4/15)	Not applicable

Data are presented as median (IQR) or n (%). *P* values were calculated using the Mann–Whitney *U* test for continuous variables and Fisher’s exact test for categorical variables, as appropriate. For genetic testing status, percentages are based on the number of patients tested within each group. Mutation-negative refers only to patients who underwent genetic testing and in whom no pathogenic or likely pathogenic variants were identified; patients without testing are listed separately. *P* values were not calculated for genetic testing categories due to the small sample size.

All surgically treated patients who underwent genetic testing (8/8) harbored *ABCC8*/*KCNJ11* mutations, whereas 7 of 11 tested non-surgical patients carried *ABCC8*/*KCNJ11* mutations; the remaining non-surgical patients were mutation-negative (2/11 among those tested) or not tested (4/15 overall) (**Table 2**).

### 5.2 Exploratory analysis of clinical factors associated with surgical intervention

**Figure 2:**
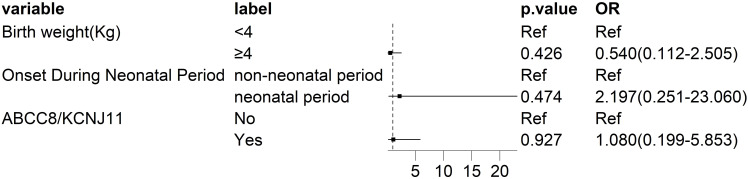
Exploratory clinical characteristics descriptively observed among patients selected for surgical management at a single center. This forest plot shows results from an exploratory Firth penalized logistic regression describing clinical and genetic characteristics observed among children with diffuse congenital hyperinsulinism (DCHI) who were selected for surgical management at a single institution (*n* = 27). Odds ratios (ORs) and 95% confidence intervals (CIs) are shown; the vertical line (OR = 1.0) indicates the null effect. Neonatal onset DCHI (0–28 days) was more frequently observed among patients selected for surgical management (OR = 2.197; 95% CI: 0.251–23.060; *P* = 0.474); however, the wide confidence interval reflects limited statistical precision. Birth weight ≥4 kg (OR = 0.540; 95% CI: 0.112–2.505; *P* = 0.426) and *ABCC8*/*KCNJ11* mutation status (OR = 1.080; 95% CI: 0.199–5.853; *P* = 0.927) did not show statistically significant associations. Surgical intervention and the resulting odds ratios reflect clinician-driven decisions within this institution and should not be interpreted as biological markers or causal predictors. All findings are descriptive and exploratory.

Exploratory Firth penalized logistic regression was applied to show how selected clinical features—such as age at onset, birth weight, and ABCC8/KCNJ11 mutation status—were considered in surgical decisions at our institution. Neonatal-onset DCHI was observed more frequently among children undergoing surgery, but these findings are descriptive and do not imply causation or predictive value (**Figure 2**).

In the Firth model, neonatal onset showed a numerically higher odds ratio of surgical intervention (OR = 2.197; 95% CI: 0.251–23.060), though the confidence interval was wide and did not suggest a clear effect. Neither birth weight ≥4 kg nor the presence of *ABCC8*/*KCNJ11* mutations showed clear influence on surgical selection.

### 5.3 Clinical characteristics and outcomes of 12 surgically treated patients with DCHI

Twelve patients underwent surgical intervention: near-total pancreatectomy (NTP, *n* = 6) or subtotal pancreatectomy (STP, *n* = 6). Most patients, 91.7% (11/12), exhibited neonatal onset, with a median age at surgery of 2.0 months (IQR: 1.7–16.0 months; range: 1.2–24.0 months). The median postoperative follow-up duration for the surgical group was 2.9 years (range: 2.0–8.8 years; **Table 3**), providing context for metabolic outcomes such as hypoglycemia remission and PD.

**Table 3:** Clinical characteristics of 12 surgically treated patients with DCHI.

Case	Onset (d)	BW (kg)	Gene mut¹	lesion	Surgery age (mo)	Proc (resection %)	Pre-op therapy (mo)	Follow-up (yr)	Surgery delay (d)	Outcome
1	1	3.99	Not tested	Diffuse	2.0	NTP (95%)	0.8	8.8	7	Remission
2	2	4.00	*KCNJ11*: c.413T>A (p.Val138Glu), heterozygous (p)	Diffuse	2.0	STP (90%)	1.8	8.8	5	Remission
3	0	4.40	*ABCC8*: c.3124_3126del (p.Thr1042Glnfs*75) (m)/c.2832_2833insA (p.Glu945Argfs*25) (p), comp het	Diffuse	23.0	STP (90%)	22.2	2.0	10	Persistent hypoglycemia
4	0	4.80	Not tested	Diffuse	24.0	NTP (95%)	23.9	4.0	8	Persistent hypoglycemia
5	460	2.70	Not tested	Diffuse	18.0	NTP (95%)	1.2	6.3	10	postoperative diabetes
6	1	4.80	*ABCC8*: c.1887delC (p.Thr630Hisfs*17) (m)/c.4310G>A (p.Arg1437Gln) (p), comp het	Diffuse	10.0	NTP (95%)	9.8	7.2	7	Remission
7	0	5.08	*ABCC8:* c.4252C>T (p.Arg1418Cys), heterozygous (p)	Diffuse	1.6	NTP (95%)	1.0	2.9	7	Remission
8	2	5.04	Not tested	Diffuse	2.0	STP (90%)	1.8	2.7	6	Persistent hypoglycemia
9	0	4.63	*ABCC8*: c.331G>A (p.Gly111Arg) (p)/c.1817 + 2T>C (IVS12) (m), comp het	Diffuse	2.0	STP (90%)	1.5	2.0	6	Persistent hypoglycemia
10	17	5.60	*ABCC8:* c.4400C>T (p.Pro1467Leu), homo (p+m)	Diffuse	1.2	NTP (95%)	1.0	2.0	2	Persistent hypoglycemia
11	0	3.99	*ABCC8*: c.4451G>A (p.Gly1484Glu), d	Diffuse	2.3	STP (92%)	1.9	2.8	2	Persistent hypoglycemia
12	0	4.28	*KCNJ11*: c.400G>C (p.Gly134Arg), d	Diffuse	1.5	STP (85%)	1.0	2.4	6	Remission

1. Genetic variants are shown in HGVS nomenclature, with inheritance indicated as paternal (p), maternal (m), *de novo* (d), homozygous (homo), or compound heterozygous (comp het).

2. Lesion: DCHI type confirmed by ^18^F–L–DOPA PET/CT.

3. Surgical procedures (NTP/STP) and outcome definitions are consistent with those in **Supplementary Table 1**.

4. Pre-op therapy: Duration of medical therapy before surgery (mo).

5. Surgery delay: Interval between meeting surgical criteria and performing surgery (days).

6. Follow-up (yr): Calculated as the interval from the date of surgery to the latest follow-up or clinical assessment.

7. Case 3 was monitored for 2 years post-surgery before being lost to contact; the reported outcome reflects his clinical status at the last evaluation.

Postoperatively, 41.7% of patients achieved complete remission, while the remaining patients experienced either persistent hypoglycemia or PD, which were analyzed as distinct outcomes. Persistent hypoglycemia was recorded in 50.0% (6/12) of patients; notably, glycemic control remained clinically stable under individualized medical management, including dietary modification and low-dose diazoxide. No recurrent severe hypoglycemic episodes requiring hospitalization occurred during long-term follow-up. PD developed in 8.3% (1/12) of patients, 6.3 years after surgery, with no additional cases observed during follow-up (**Table 3**).

**Figure 3:**
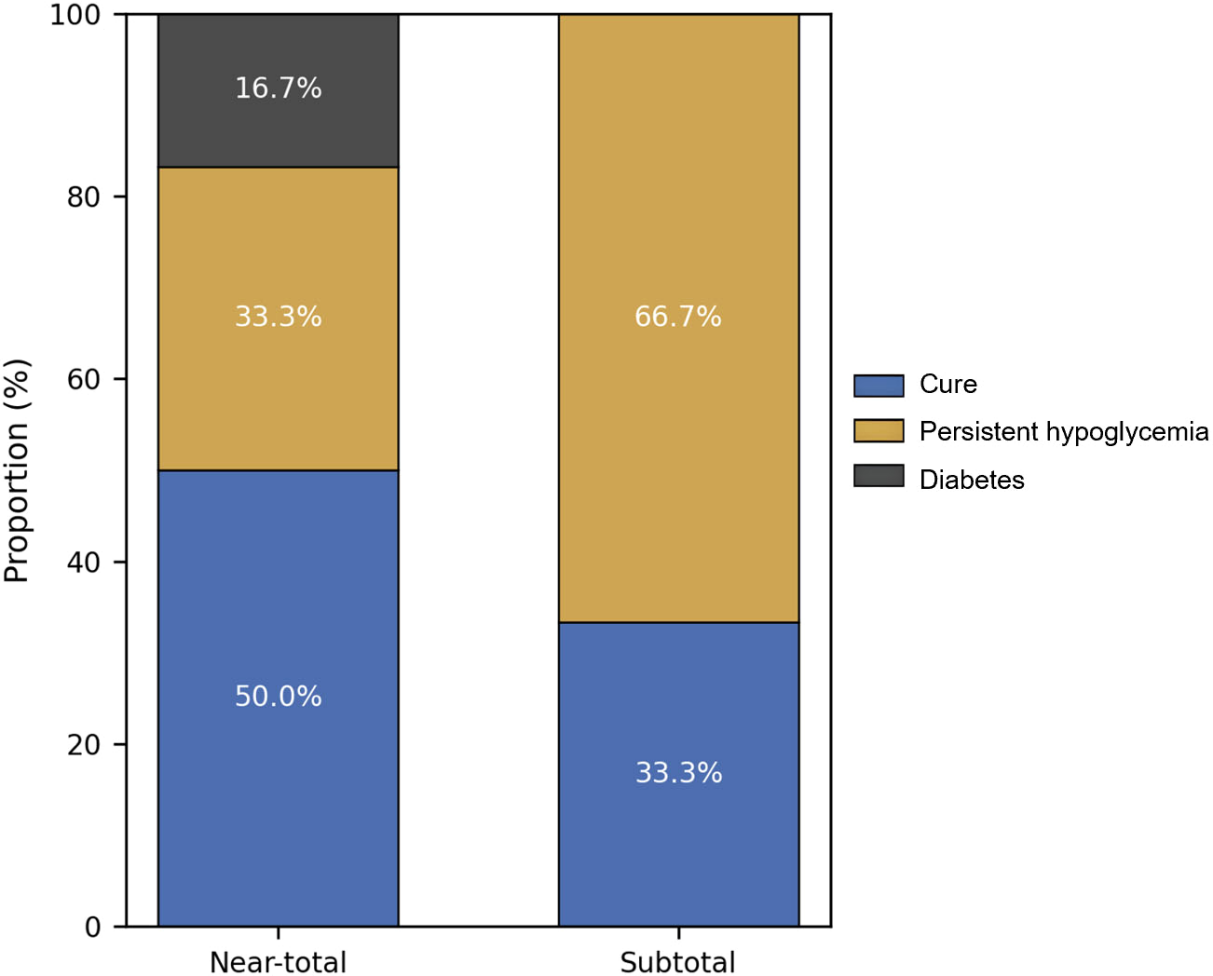
Postoperative outcome distribution of patients with DCHI undergoing different surgical procedures. This figure shows the distribution of postoperative metabolic outcomes among 12 children with diffuse congenital hyperinsulinism (DCHI) undergoing two surgical strategies: near-total pancreatectomy (NTP, 95–98% resection; n = 6) and subtotal pancreatectomy (STP, 80–94% resection; n = 6). Outcomes include (i) complete remission, (ii) persistent or recurrent hypoglycemia, and (iii) postoperative diabetes (PD). The NTP group showed a numerically higher remission rate (50.0% vs. 33.3%) and a higher observed frequency of PD (16.7% vs. 0%) compared with the STP group. However, these differences did not reach statistical significance (Fisher’s exact test, P > 0.05), likely reflecting the limited sample size. These findings are descriptive and intended to illustrate potential clinical trade-offs between glycemic control and long-term metabolic complications across different resection extents.

In the comparison of surgical extents, remission was observed in 50.0% (3/6) of NTP patients and 33.3% (2/6) of STP patients, while PD occurred in 16.7% (1/6) versus 0% (0/6), respectively (**Table 4**). Given the very small sample size, these comparisons are descriptive and intended to illustrate trends rather than infer statistical significance (**Figure 3**).

**Table 4:** Exploratory comparison of metabolic outcomes between near-total and subtotal pancreatectomy in DCHI (*n* = 12).

Variables	NTP (*n* = 6)	STP (*n* = 6)	*P* value
Remission (off medication, euglycemia)	3 (50.0%)	2 (33.3%)	1.000
Persistent hypoglycemia	2 (33.3%)	4 (66.7%)	0.567
Postoperative diabetes	1 (16.7%)	0 (0%)	1.000

Data are presented as *n* (%). Comparisons were performed using Fisher’s exact test. Due to the small sample size (*n* = 12, *n* = 6 per subgroup), these comparisons are exploratory and descriptive, and the lack of statistical significance (P > 0.05) should be interpreted with caution. Given the extremely small sample size, *P* values are presented for completeness only and do not indicate equivalence or absence of clinically relevant differences. Postoperative outcomes are defined as in **Supplementary Table 1**. Surgical procedures (NTP vs. STP) were classified according to the estimated extent of resection recorded in the operative reports.

When stratified by surgical timing (<1 year vs. ≥1 year), remission was more common in younger patients: 55.6% (5/9) vs. 0% (0/3); these observations are descriptive and exploratory.

All patients received preoperative medical therapy for a median of 1.7 months (IQR: 1.0–1.9 months; range: 0.8–24.3 months), and the median interval from meeting surgical criteria to intervention was 6.5 days (range: 2–10 days), reflecting timely surgical intervention.

For completeness and to clarify the contrast with surgically treated patients, outcomes in the non-surgical group are briefly summarized. Long-term follow-up of non-surgical patients demonstrated that none experienced recurrent or frequent hypoglycemic episodes requiring hospitalization or therapy escalation. All maintained stable glycemic control with medical therapy alone, and therefore did not meet criteria for surgery.

### 5.4 Neurodevelopmental outcomes and long-term neurological risk in 27 patients with DCHI

#### 5.4.1 Neurodevelopmental outcomes at last follow-up (available-case analysis, *n* = 25)

Long-term neurodevelopmental outcomes were available for 25 of 27 patients (surgical group: *n* = 11; non-surgical group: *n* = 14), with one patient lost to follow-up in each group. These two patients were excluded from the cross-sectional analysis of neurological outcomes.

Favorable neurological outcomes were recorded in 64% (16/25) of patients, whereas 36% (9/25) experienced neurological sequelae, primarily presenting as psychomotor developmental delay or epilepsy. The incidence of neurological sequelae showed no apparent difference between surgical and non-surgical groups 27.3% (3/11) vs. 42.9% (6/14); *P* = 0.678 (**Table 1**).

#### 5.4.2 Time-to-event analysis of neurological outcomes (Kaplan–Meier analysis, *n* = 27)

**Figure 4:**
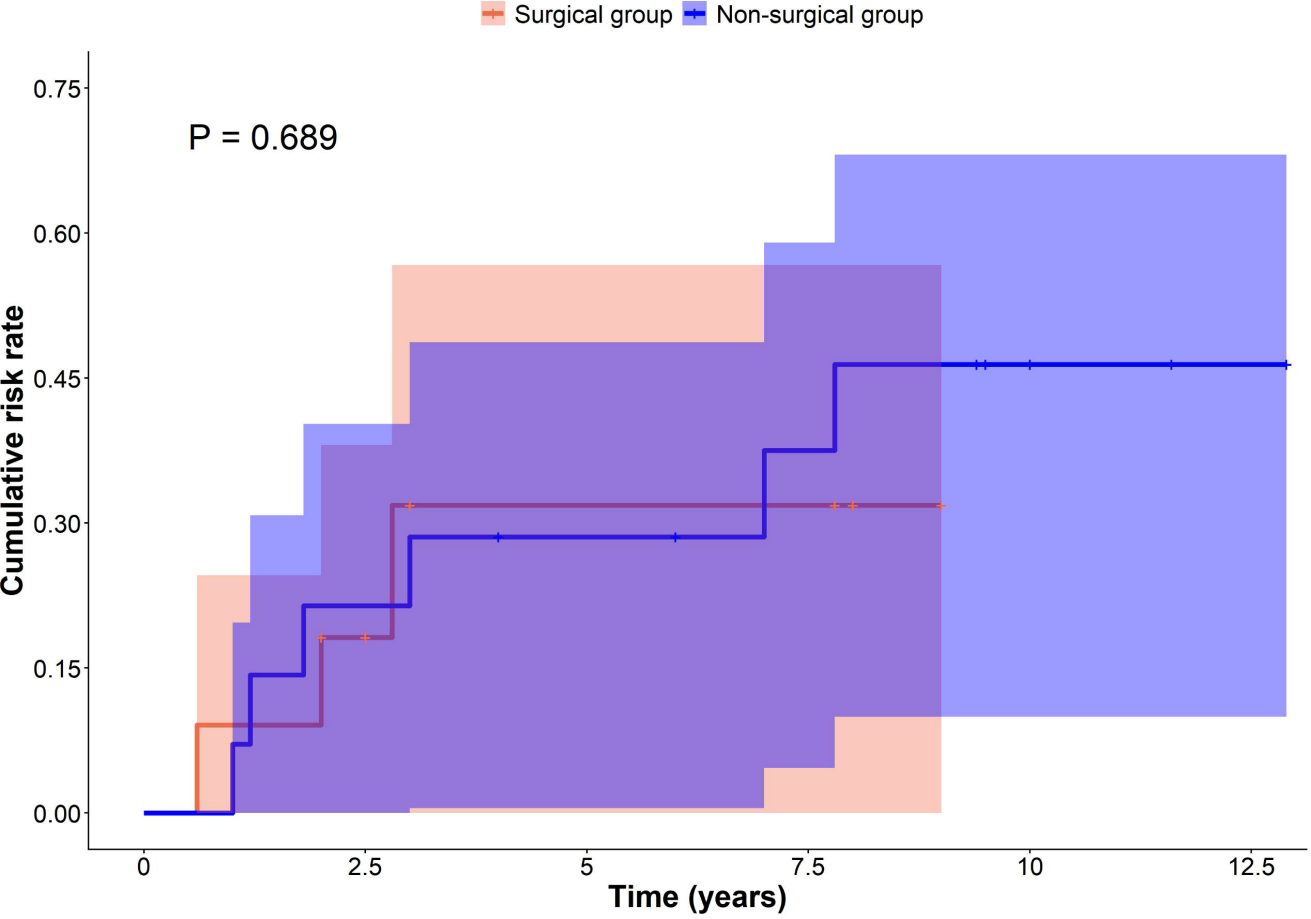
Descriptive Kaplan–Meier curves of cumulative neurological sequelae in patients with diffuse congenital hyperinsulinism (DCHI) treated surgically (*n* = 12) or medically (*n* = 15). Censored observations are indicated by tick marks; patients lost to follow-up were treated as censored. Shaded areas represent 95% confidence intervals. The y-axis represents the cumulative probability of neurological sequelae (%). This Kaplan–Meier analysis is presented descriptively to illustrate long-term neurological trends over follow-up. Given the limited sample size and heterogeneity of assessment modalities, this analysis was intended to describe long-term neurodevelopmental trends rather than to establish definitive group differences. The log-rank *P* value is provided for reference only and should not be interpreted as confirmatory evidence.

For time-to-event analysis, individual follow-up for surgically treated patients ranged from 2.0 to 8.8 years (median 2.9 years), while follow-up for the entire cohort ranged from 2–13 years. Two patients lost to follow-up were treated as censored. Kaplan–Meier (KM) analysis was applied descriptively to visualize temporal patterns in cumulative neurological risk rather than to support formal inferential comparisons. The KM curves demonstrated no apparent separation in cumulative neurological risk between surgical and non-surgical groups (log-rank *P* = 0.689; **Figure 4**), and this P value should be interpreted cautiously given the exploratory design and limited sample size. Given the small and balanced number of censored cases, their impact on the comparison is expected to be minimal.

Neurological outcomes appeared more frequently observed among patients with earlier hypoglycemia exposure; these observations are descriptive and hypothesis-generating, and should not be interpreted as evidence of time-dependent risk differences or treatment effects.

## 6 Discussion

### 6.1 Genetic background and clinical features

The exploratory regression-based associations observed in this study likely reflect clinician-driven selection for surgery rather than biological predictors of surgical necessity. In our single-center cohort, *ABCC8*/*KCNJ11* mutations were more prevalent among patients selected for surgery, illustrating institutional practice patterns in surgical decision-making, consistent with prior reports (27–29). These mutations impair K_ATP_ channel function and have been reported to be frequent in patients with diazoxide-refractory hypoglycemia.

Consistent with global observations (13, 30, 31), neonatal-onset CHI appeared more common among surgically treated patients in our cohort. However, this observation is descriptive and reflects how clinical features are weighed in real-world practice rather than identifying predictors of surgical indication. Mechanistically, immature β-cell regulation in the neonatal period may exacerbate hyperinsulinemia in the presence of pathogenic K_ATP_ mutations (32, 33). Additionally, non-K_ATP_ metabolic genes, such as *GCK*, may contribute to atypical DCHI presentations through low-level somatic mutations, further expanding the spectrum of phenotypic and therapeutic heterogeneity (34).

The exploratory Firth penalized logistic regression was performed to illustrate institutional decision-making patterns rather than to identify biological predictors of surgical necessity. In our single-center cohort, *ABCC8/KCNJ11* mutations were more prevalent among surgically treated patients, consistent with prior reports, reflecting clinician-driven selection rather than causal effects. Neonatal-onset CHI also appeared more frequent in the surgical group, underscoring how clinical features inform real-world practice.

The wide confidence intervals and lack of statistically meaningful associations further highlight the heterogeneity of clinical presentations and the reliance on clinician judgment, institutional experience, and contextual factors. Both the exploratory regression and Kaplan–Meier analyses were performed descriptively to capture temporal trends and institutional decision-making patterns, without making formal inferential or predictive claims.

### 6.2 Efficacy, risks, and therapeutic balance of different pancreatic resection strategies in 12 patients with DCHI

Among surgically treated patients, a numerically higher remission rate was observed among patients undergoing near-total pancreatectomy (NTP) compared with those undergoing subtotal pancreatectomy (STP). However, this pattern was accompanied by a higher frequency of long-term PD, illustrating the fundamental therapeutic trade-off between glycemia control and preservation of endocrine function.

Our observations are consistent with outcomes from major global centers, including CHOP and European multicenter cohorts (16, 17, 35–37). Recent Chinese data involving 23 predominantly DCHI patients demonstrated similar postoperative hypoglycemia control (~70%) and PD rates (~20%) (38). Although differences between surgical subgroups were not statistically significant in our cohort (*n* = 6 per group), these trends provide descriptive insight into the balance of efficacy and long-term metabolic risk.

Previous studies indicate that NTP provides maximal reduction in insulin secretion but carries a higher long-term risk of PD, whereas STP preserves more pancreatic tissue but may be linked to persistent hypoglycemia, occasionally necessitating reoperation (11, 17, 35–40). Long-term follow-up from a French multicenter cohort of 105 patients showed that PD risk continues to increase over time regardless of the extent of resection, underscoring the importance of ongoing metabolic monitoring (17). Early postoperative complications also differ between procedures and should be considered when planning surgery.

Therefore, the extent of pancreatectomy may be considered on an individual basis, with attention to the observed balance between short-term glycemic stability and long-term metabolic morbidity (23). In conclusion, these findings inform clinical decision-making by highlighting observed therapeutic trends.

### 6.3 Long-term dynamic risk of neurodevelopmental outcomes and optimal timing of surgical intervention in 27 patients with DCHI

No significant difference was observed in the incidence of neurodevelopmental sequelae between surgical and non-surgical groups (27.3% vs. 42.9%; *P* = 0.678). These results, based on available-case data, indicate that surgical intervention *per se* is not the sole determinant of neurological status. Patients lost to follow-up were treated as censored in Kaplan–Meier analyses, which were applied to illustrate cumulative neurological risk (log-rank *P* = 0.689) rather than to infer precise time-dependent outcomes. Within our cohort, treatment modality was not observed to clearly influence cumulative neurological risk.

These observations suggest that early and prolonged hypoglycemia exposure, rather than treatment modality per se, may have a greater impact on long-term neurodevelopmental outcomes. Timely recognition and effective management of hypoglycemia remain critical for safeguarding neurological function. In our cohort, once surgical criteria were met, the interval to intervention was short (median 6.5 days), making surgical delay unlikely to significantly affect outcomes. However, due to the retrospective design, the total duration and severity of early hypoglycemia prior to referral could not be fully quantified.

Our findings are consistent with prior reports: Muukkonen et al. reported that children with persistent CHI exhibited specific neurocognitive deficits compared with population norms, consistent with hypoglycemia-related brain injury (41); Ludwig and Mohnike observed a high prevalence of neurodevelopmental abnormalities in a large CHI cohort, highlighting that neurologic impairment frequently presents early in the disease course (42); and Laimon et al. demonstrated that prolonged hypoglycemia and delayed diagnosis were associated with increased risk of neurological sequelae, independent of treatment modality (26). While these observations are descriptive and exploratory in nature, they suggest that early and prolonged hypoglycemia exposure, rather than surgical timing or extent, may have the greatest impact on long-term neurodevelopmental outcomes. The retrospective design and small sample size limit causal inference, and the potential influence of surgical timing and extent remains an important area for future prospective studies.

Formal preoperative neurodevelopmental assessments were not systematically available; thus, neurological impairment may have occurred before surgery. While timely surgical intervention may benefit neonatal-onset or diazoxide-unresponsive cases (5, 43–45), our findings suggest that the timing of surgery may be more pivotal for neuroprotection than the choice between NTP and STP.

Surgical intervention may mitigate neurodevelopmental risk by reducing the frequency and severity of glycemic fluctuations. No direct statistical association between surgery and neurodevelopmental outcomes (measured via DQ/IQ scores) was observed. Consequently, when medical therapy fails, timely surgery remains a critical strategy to prevent ongoing brain injury in medically refractory patients.

### 6.4 Role and limitations of medical therapy in DCHI

Medical therapy, particularly diazoxide, remains the first-line treatment for DCHI, with surgery reserved for cases refractory to medical management, in line with international consensus (6, 7, 45). Emerging therapies, including long-acting or novel glucagon analogs, show promise in reducing intravenous glucose requirements (46). mTOR inhibitors have demonstrated limited efficacy and potential side effects, specifically, sirolimus is not recommended as a standard therapy in international guidelines, but may be considered in selected cases with careful monitoring (6, 8, 47–51). Evidence from Chinese cohorts supports the effectiveness of early, appropriately titrated medical management in monogenic CHI (7).

### 6.5 Study limitations and future directions

Several limitations warrant consideration. First, given the descriptive nature of this retrospective cohort study, this work did not aim to infer causal relationships or comparative effectiveness. In particular, exploratory regression analyses were applied to depict institutional decision-making patterns rather than to establish predictors of surgical necessity or disease biology. Observed trends, such as the higher remission rates in patients undergoing surgery prior to 1 year of age, are consequently reported descriptively. The relatively small sample size, inherent to this rare condition, may limit the statistical power to detect subtle differences between treatment subgroups.

Second, the retrospective design led to incomplete biochemical and genetic data, heterogeneous neurodevelopmental follow-up, and an inability to precisely quantify cumulative preoperative hypoglycemia exposure. Neurodevelopmental outcomes were assessed using a combination of standardized in-person evaluations (e.g., Gesell, WISC) and milestone-based telephone interviews at variable ages, introducing potential ascertainment bias. This pragmatic dichotomization facilitated longitudinal comparison but inevitably limited the granularity and interpretability of neurodevelopmental outcomes.

Third, the extent of pancreatic resection was determined by subjective intraoperative estimation rather than objective volumetric quantification. Although this may introduce potential classification variability near the 95% resection threshold, it reflects real-world clinical practice and aligns with methodologies used in major CHI surgical cohorts.

Lastly, variable follow-up duration may have led to an underestimation of long-term PD risk. These limitations highlight the need for larger, multicenter studies with standardized assessments, longitudinal metabolic monitoring, and objective quantification of surgical extent.

Despite these constraints, this study provides the largest single-center description of Chinese children with DCHI undergoing pancreatectomy. Our findings offer insights to guide clinical decision-making, though future multicenter prospective studies with standardized metabolic and neurodevelopmental assessments are warranted to optimize long-term outcomes.

## 7 Conclusion

Pancreatic resection, including subtotal and near-total procedures, is commonly employed for medically refractory DCHI. Neonatal-onset disease was frequently observed among surgically treated patients, reflecting institutional selection rather than a validated predictor.

Near-total pancreatectomy was associated with greater glycemic stability but higher long-term PD, whereas subtotal pancreatectomy had more persistent/recurrent hypoglycemia. These observations are descriptive and hypothesis-generating, requiring validation in larger multicenter studies. Long-term neurological outcomes appeared more influenced by timing and severity of early hypoglycemia than surgical approach, emphasizing early diagnosis and prompt glycemic management.

## Data Availability

The original contributions presented in the study are included in the article/**Supplementary Material**. Further inquiries can be directed to the corresponding author.
