# Supplementary material for: Single-center experience description of surgical management of diffuse congenital hyperinsulinism in a pediatric non-current cohort
Source: Front Endocrinol (Lausanne). 2026 Mar 23;17:1760749. doi: 10.3389/fendo.2026.1760749 (PMC13050746; doi:10.3389/fendo.2026.1760749)
Supplement: Supplementary file 1 [file Table1.docx]

Supplementary Table S1. Definitions of surgical procedures and postoperative outcomes

| Term | Definition |
| --- | --- |
| Subtotal pancreatectomy (STP) | Surgical intent to resect approximately 80–94% of pancreatic tissue, based on operative reportsᵃ |
| Near-total pancreatectomy (NTP) | Surgical intent to resect approximately 95–98% of pancreatic tissue, leaving a small rim along the bile duct or duodenumᵃ |
| Postoperative remission | Discontinuation of all glucose-lowering medications with fasting glucose levels of 3.88–5.6 mmol/Lᵇ |
| Persistent postoperative hypoglycemia | Continued requirement for medication or supplemental feeding to maintain euglycemiaᶜ |
| Postoperative diabetes | Requirement for long-term insulin therapyᶜ |

**Abbreviations:** STP, subtotal pancreatectomy; NTP, near-total pancreatectomy.

**Notes:
^a^** Definitions of STP and NTP are based on operative reports and consistent with major international CHI surgical cohorts and guidelines (CHOP, Beltrand, Adzick) (6, 23, 24).
**^b^** Postoperative remission definition adapted from references (6)

**^c^** Persistent postoperative hypoglycemia and postoperative diabetes are defined according to criteria used in this study. Persistent postoperative hypoglycemia and postoperative diabetes represent distinct postoperative metabolic outcomes and were not combined into a single composite endpoint in outcome analyses.

Supplementary Table S2. Neurodevelopmental assessments, follow-up methods, and outcomes in the study cohort

| Case No. | Surgical treatment  (Yes/No) | Assessment modality | Tool used | Age at assessment / last follow-up (years) | Key findings / Score | Neurological sequelae (Yes/No) |
| --- | --- | --- | --- | --- | --- | --- |
| 1 | NO | In-person | Gesell | 1.0 | Language DQ=65 | Yes |
| 2 | NO | Telephone follow-up | Milestone-based | 10.0 | Normal | NO |
| 3 | NO | In-person | WISC | 7.0 | FSIQ=68 | Yes |
| 4 | NO | Telephone follow-up | Milestone-based | 10.0 | Normal | NO |
| 5 | Yes | Telephone follow-up | Milestone-based | 9.0 | Normal | NO |
| 6 | NO | Telephone follow-up | Milestone-based | 9.5 | Normal | NO |
| 7 | Yes | Telephone follow-up | Milestone-based | 9.0 | Normal | NO |
| 8 | Yes | Lost to  follow-up | ND | ND | ND | Lost to  follow-up |
| 9 | Yes | In-person | Gesell | 2.0 | GDD(Total DQ=62;all domains < 70) | Yes |
| 10 | NO | Telephone follow-up | Milestone-based | 11.6 | Normal | NO |
| 11 | NO | In-person | WISC | 7.8 | FSIQ=66 | Yes |
| 12 | NO | In-person | WISC | 9.4 | FSIQ = 99 | NO |
| 13 | NO | Telephone follow-up | Milestone-based | 1.2 | Delayed speech | Yes |
| 14 | NO | Telephone follow-up | Milestone-based | 12.9 | Normal | NO |
| 15 | Yes | In-person | WISC | 7.8 | FSIQ=96 | NO |
| 16 | NO | Lost to follow-up | ND | ND | ND | Lost to follow-up |
| 17 | NO | In-person | Gesell | 3.0 | Language DQ=71 | Yes |
| 18 | NO | In-person | Gesell | 1.8 | Language DQ=68 | Yes |
| 19 | Yes | In-person | WISC | 8.0 | FSIQ=102 | NO |
| 20 | NO | Telephone follow-up | Milestone-based | 6.0 | Normal | NO |
| 21 | Yes | In-person | Gesell | 3.0 | Total DQ = 99 | NO |
| 22 | Yes | In-person | Gesell | 2.8 | GDD(Total DQ = 62; all domains < 70) with Epilepsy | Yes |
| 23 | NO | In-person | Gesell | 4.0 | Total DQ = 100 | NO |
| 24 | Yes | In-person | Gesell | 2.2 | Total DQ = 90 | NO |
| 25 | Yes | In-person | Gesell | 2.1 | Total DQ = 105 | NO |
| 26 | Yes | In-person | Gesell | 0.7 | Gross Motor  DQ=69 | Yes |
| 27 | Yes | In-person | Gesell | 2.5 | Total DQ = 98 | NO |

**Notes:**

1. **Assessment Modalities:** In-person neurodevelopmental evaluations were conducted by trained specialists using the Gesell Developmental Schedules (n = 12) or the Wechsler Intelligence Scale for Children (WISC; n = 4). For patients unable to attend in-person assessments (n = 9), structured telephone interviews focusing on age-appropriate developmental milestones were performed by experienced pediatricians.
2. **Definitions:** Neurodevelopmental delay was defined as a developmental quotient (DQ) or intelligence quotient (IQ) < 70, or clinically evident delay in achieving age-appropriate milestones. “Normal” indicates attainment of expected developmental milestones without evidence of delay.
3. **Age at Assessment (Time-to-Event):** For patients with neurological sequelae, age at assessment represents the time point at which impairment was first clinically identified (event time). For patients without sequelae, it represents the age at the most recent effective follow-up confirming normal development and was treated as censored time in Kaplan–Meier analyses.
4. **Missing data:** ND indicates not determined. Outcomes were not imputed for patients lost to follow-up (n = 2), and analyses were conducted using an available-case approach.
5. **Assessment frequency:** For each patient, the most recent valid neurodevelopmental assessment was used for analysis. Repeated formal assessments were not systematically available and therefore were not analyzed longitudinally.
